# Identification of Novel SHOX Target Genes in the Developing Limb Using a Transgenic Mouse Model

**DOI:** 10.1371/journal.pone.0098543

**Published:** 2014-06-02

**Authors:** Katja U. Beiser, Anne Glaser, Kerstin Kleinschmidt, Isabell Scholl, Ralph Röth, Li Li, Norbert Gretz, Gunhild Mechtersheimer, Marcel Karperien, Antonio Marchini, Wiltrud Richter, Gudrun A. Rappold

**Affiliations:** 1 Department of Human Molecular Genetics, Heidelberg University Hospital, Heidelberg, Germany; 2 Division of Experimental Orthopaedics, Orthopaedic University Hospital, Heidelberg, Germany; 3 Medical Research Center (ZMF), Medical Faculty Mannheim at Heidelberg University, Mannheim, Germany; 4 Institute of Pathology, Heidelberg University Hospital, Heidelberg, Germany; 5 Department of Developmental Bioengineering, University of Twente, Enschede, The Netherlands; 6 German Cancer Research Center (DKFZ), Heidelberg, Germany; University of Massachusetts Medical, United States of America

## Abstract

Deficiency of the human short stature homeobox-containing gene (*SHOX*) has been identified in several disorders characterized by reduced height and skeletal anomalies such as Turner syndrome, Léri-Weill dyschondrosteosis and Langer mesomelic dysplasia as well as isolated short stature. SHOX acts as a transcription factor during limb development and is expressed in chondrocytes of the growth plates. Although highly conserved in vertebrates, rodents lack a *SHOX* orthologue. This offers the unique opportunity to analyze the effects of human *SHOX* expression in transgenic mice. We have generated a mouse expressing the human *SHOXa* cDNA under the control of a murine *Col2a1* promoter and enhancer (*Tg*(*Col2a1-SHOX*)). *SHOX* and marker gene expression as well as skeletal phenotypes were characterized in two transgenic lines. No significant skeletal anomalies were found in transgenic compared to wildtype mice. Quantitative and *in situ* hybridization analyses revealed that *Tg*(*Col2a1-SHOX*), however, affected extracellular matrix gene expression during early limb development, suggesting a role for *SHOX* in growth plate assembly and extracellular matrix composition during long bone development. For instance, we could show that the connective tissue growth factor gene *Ctgf*, a gene involved in chondrogenic and angiogenic differentiation, is transcriptionally regulated by SHOX in transgenic mice. This finding was confirmed in human NHDF and U2OS cells and chicken micromass culture, demonstrating the value of the *SHOX*-transgenic mouse for the characterization of SHOX-dependent genes and pathways in early limb development.

## Introduction

Height is a complex trait defined by multiple biological and environmental factors that are involved in bone formation and growth. The development of the long bones is characterized by coordinated gene expression from early embryonic stages until adulthood. Disturbances in bone development can affect growth and lead to clinical consequences. The homeodomain transcription factor SHOX is involved in different human short stature syndromes (Turner syndrome, Léri-Weill dyschondrosteosis LWD [MIM 127300] and Langer mesomelic dysplasia [MIM 249700]) and isolated (idiopathic) short stature [MIM 300582] [1], [2], [3], [4], [5], [6], [7]. Mutations and deletions of the *SHOX* gene and its enhancers have been identified as etiologic for the short stature and skeletal anomalies in these disorders [8], [9], [10], [11], [12], [13]. Comprehensive case studies have shown that *SHOX* defects have also been identified in the more common nonsyndromic (isolated) forms of short stature with a prevalence of 5–17% in geographically different populations [6], [12], [14]. An overdosage of *SHOX* as in patients with Triple-X or Klinefelter syndrome results in tall stature [15].

Phenotypic characteristics are variable in *SHOX*-deficient patients and include disproportional (mesomelic) short stature, shortening of the forearms as well as Madelung deformity, a skeletal abnormality of the wrist characteristic for LWD [4], [16]. Histopathological evaluation of LWD growth plates revealed a variable disruption of the architecture and an irregular chondrocyte stacking [17], and the SHOX protein was mainly detected in prehypertrophic and hypertrophic chondrocytes of fetal and childhood growth plates by immunohistochemistry [18], [19], [20]. Since clinical studies have demonstrated that growth hormone (somatropin) therapy before the onset of puberty effectively ameliorates the short stature in *SHOX*-deficient patients [21], a somatropin-based therapy is proposed in affected individuals.

Despite the high clinical relevance of *SHOX* mutations, surprisingly little is known about the molecular mechanisms that are governed by *SHOX* deficiency. This is mainly due to the limited availability of patient tissue samples (growth plate material) and the lack of cellular systems that reliably express *SHOX* endogenously at sufficiently high levels [22]. Mice do not have a *SHOX* orthologue, thus a knock-out model cannot be generated. Since the vast majority of genes that govern early developmental processes are highly conserved between human and mouse [23], characterization of genes that are divergent between the two species has not attracted much attention. SHOX has been shown to act as both a transcriptional activator and repressor of target genes [8], [20], [24], [25], [26]. Functional studies have also shown that overexpression of the SHOX protein can induce growth arrest and apoptosis, suggesting that SHOX may regulate chondrocyte hypertrophy by inducing apoptosis [19].

The clinical relevance of *SHOX* in short stature prompted us to generate a transgenic mouse to study the effect of the human *SHOX* gene during early chondrogenesis. While the phenotypic features are sparse in these animals, we demonstrate that *Ctgf*, among other genes, is regulated by SHOX in transgenic mice as well as in human and chicken cell cultures. In addition, microarray and molecular analyses revealed that the *SHOX*-transgene can effectively regulate genes important in early processes during limb formation.

## Materials and Methods

### Animals and genotyping

All animal experiments were conducted according to German animal protection laws and approved by the regional board of Baden Württemberg (permission No. 35–9185.81/G–64/05 and A-30/09). To express *SHOX* (genomic coordinates according to GRCh37: X:585,078-620,145) in mouse limbs, the *SHOXa* cDNA (CCDS14107.1) was cloned into the murine expression vector p1757 including the rat *Col2a1* promoter (1 kb), a Globin splicing sequence (640 bp) and the *Col2a1* enhancer (1.4 kb) [27], [28], [29] and a SV40 polyadenylation signal from pGL3 Basic (Promega). The construct (p1757 SHOX) was linearized with *AgeI* and microinjected into pronuclei of fertilized C57BL/6 x DBA/2 hybrid eggs to generated transgenic mice. Founders were identified by extraction of genomic DNA from tails followed by PCR using primers SHOX1 and XHO_REV (1-409 of the *SHOXa* cDNA) and SHOX_ECORI_FOR and LUMIOSHOXCTER_REV (242-TGA of the *SHOXa* cDNA). Southern Blot was carried out according to standard procedures with a probe spanning nucleotides 1-409 to confirm the integration of the transgene at a single locus. Primer sequences are included in the Table S2 in File S1.

### Limb preparation and RNA samples

Limbs of wildtype and transgenic littermates at E10.5-E14.5 were dissected and frozen in liquid nitrogen. RNA was isolated using the RNeasy Kit (Qiagen), following homogenization using a PT1300 D polytron (Kinematica). DNA was hydrolyzed using the RNAse-free DNAse Kit (Qiagen). RNA yield was measured using a NanoDrop 2000 spectrophotometer (Nanodrop technologies) and quality-checked on agarose gels. For microarray analysis, RNA from 2-4 E12.5 wildtype and transgenic littermates was pooled and the quality-checked on a 2100 Bioanalyzer (Agilent).

### *In vitro* transcription and quantitative RT-PCR

*In vitro* transcription of 1 µg RNA was performed using the Superscript II First Strand Synthesis System for RT-PCR (Invitrogen). qRT-PCR was carried out using the Applied Biosystems 7500 Real-Time PCR System and Absolute SYBR Green ROX Mix (Abgene). Each sample and the housekeeping genes were run in duplicates. Relative mRNA levels were calculated according to the delta-delta Ct method [30] by normalization to mRNA expression of the housekeeping genes *Sdha* and *Adam9*. Primer sequences are included in Table S2 in File S1.

### μCT imaging and analysis

Transgenic and wildtype littermates were anesthetized by i.p. injection of Ketamin (75 mg/kg) and Domitor (1 mg/kg) at the age of 4 (P28–30), 12 (P84–86) and 24 weeks (P168–170). Microcomputed tomography analyses on tibiae and femora of narcotized mice was performed using a Skyscan 1076 *in vivo* scanner (Skyscan, Antwerp, Belgium) at a resolution of 17.7 µm/pixel with an 0.5 mm aluminium filter. A source voltage of 48 kV, current of 200 µA, exposure time of 320 ms and a rotation step of 0.6 degree were used. Reconstructions (NRecon, Skyscan, Antwerp, Belgium) were made using an under-sampling factor of 1, a threshold for defect pixel mask of 30%, a beam hardening correction factor of 100%, minimum of 0.0061 and maximum of 0.0674 for CS to image conversion. Length of long bones and cortical thickness were measured manually using ruler tool function (CTAn, Skyscan, Antwerp, Belgium). Equal anatomical bone markers were used for reproducibility. For quantitative analysis of bone volume (BV) and bone mineral density (BMD) a region of interest was chosen that included the total bone and thresholds of 68-255 were used for binarisation. For BMD measurement mice were euthanized at the age of 24 weeks, legs were prepared and scanned again in water. Phantoms with known densities of 0.25 and 0.75 g/cm^3^ and water were scanned for houndsfield unit calibration. Statistical analyses were carried out using Student's t-test and GraphPad Prism 5 software.

### Microarray analysis

Gene expression profiling was performed using GeneChip Mouse Genome 430.2 from Affymetrix (Santa Clara, CA, USA). Duplicate Arrays were done for each genotype (transgene or wildtype). cDNA, cRNA synthesis and hybridization to arrays were performed according to the recommendations of the manufacturer. Microarray data were submitted to NCBI GEO, sample number GSE47902. Microarray data was analyzed based on ANOVA using the software package JMP Genomics, version 4.0 (SAS Institute, Cary, NC, USA). Values of perfect-matches were log transformed, quantile normalized and fitted with log-linear mixed models, with probe_ID and genotype considered to be constant and the sample ID random. Custom CDF version 13 with Entrez gene based gene/transcript definitions (http://brainarray.mbni.med.umich.edu/Brainarray/Database/CustomCDF/genomic_curated_CDF.asp) different from the original Affymetrix probe set definitions were used to annotate the arrays. Gene Set Enrichment analysis (GSEA 2.0) was applied to reveal biological pathways modulated between sample groups. Genes were ranked according to the expression change between genotypes. All Gene Ontology terms were examined using 1000 rounds of permutation of gene sets. Pathways with absolute NES (normalized enrichment score) more than 1.7 and NP (normalized p-value) <0.02 were considered to be differentially modulated.

### The nCounter system assay

Assays were performed using 100 ng of total RNA plus reporter and capture probes for 10 genes (nanostring codeset). After over-night hybridization, sample purification and nCounter digital reading, counts for each RNA species were extracted and analyzed using a home-made Excel macro. Codesets include positive controls (spiked RNA at various concentrations) as well as negative controls (alien probes for background calculation). Background correction consisted of the subtraction of negative control average plus two SD from the raw counts. To avoid negative values, signals lower than one after correction were thresholded to one. The positive controls were used as a quality assessment. For each sample, the ratio between sample-related positive control average and the smallest positive control average was accepted when lower than 3. To select adequate normalization genes from series of candidates included in the CodeSet, the geNorm method (5) was implemented. Therefore, the geometric mean of the selected normalization genes according to geNorm was calculated and used as normalization factor. Normalized values were then compared between samples. Probe sequences are included in Table S2 in File S1.

### *In situ* hybridization

Whole-mount *in situ* hybridization using embryos fixed in 4% paraformaldehyde was performed according to standard procedures. Section *in situ* hybridisation was performed on 12 µm paraffin sections using standard protocols. Antisense riboprobe for *Ctgf* was cloned using the pSTBlue-1 AccepTor vector Kit (Novagen) with the primers Ctgf_ISH_FOR: AAA TGC TGC GAG GAG TGG GTG and Ctgf_ISH_REV: GTG CGT TCT GGC ACT GTG CGC. Antisense riboprobe for SHOX was generated from a *Bam/XhoI* fragment of pBSK SHOX, *Shox2* riboprobe was used as reported [31]. Templates for antisense *in vitro* transcription were digested and digoxigenin-labelled antisense RNA was synthesized using MEGAscript ® Kit (Ambion) as follows: SHOX: *KpnI*/Sp6; Ctgf: *BamHI*/Sp6; Ihh: *XbaI*/T7; Col10a1: *XhoI*/T3; Col2a1: *EcoRI*/T7; Fgfr3: *NdeI*,/T7; Shh: *HindIII*/T3; Runx2: *SpeI*/T7; Shox2: *SacI*/T7; Ogn: *XhoI*/T7.

### Cell culture, transfections and luciferase assays

Cells were cultivated and transfections as well as reporter gene assays were carried out as reported before [26]. Primers used for the cloning of the reporter construct are included in Table S2 in File S1.

### Electrophoretic Mobility Shift Assays (EMSA)

EMSA were carried out as described [10] using the probes sequences included in the Table S2 in File S1.

### Immunohistochemistry

Immunohistochemistry was performed on growth plate sections from a pubertal 12 years old boy (tibial growth plate) as described [19] using anti SHOX- and anti-CTGF (clone L20, Santa Cruz) antibodies at the dilution of 1∶25 and 1∶100, respectively.

### Histology

For histological examination of growth plates, femora and tibiae of wildtype and transgenic mice (24 weeks of age) were fixed in 4% formalin and decalcified in 10% EDTA. The femora and tibiae were then bisected in the middle, and paraffin embedded. Subsequently, paraffin sections were cut at 4 µm intervals in the plane of the physis. The sections were stained with hematoxylin and eosin (H&E), periodic acid-Schiff (PAS) and Masson's trichrome (MT) by standard protocols.

## Results

### Generation and expression studies of *Col2a1-SHOX*-transgenic mice

**Figure 1:**
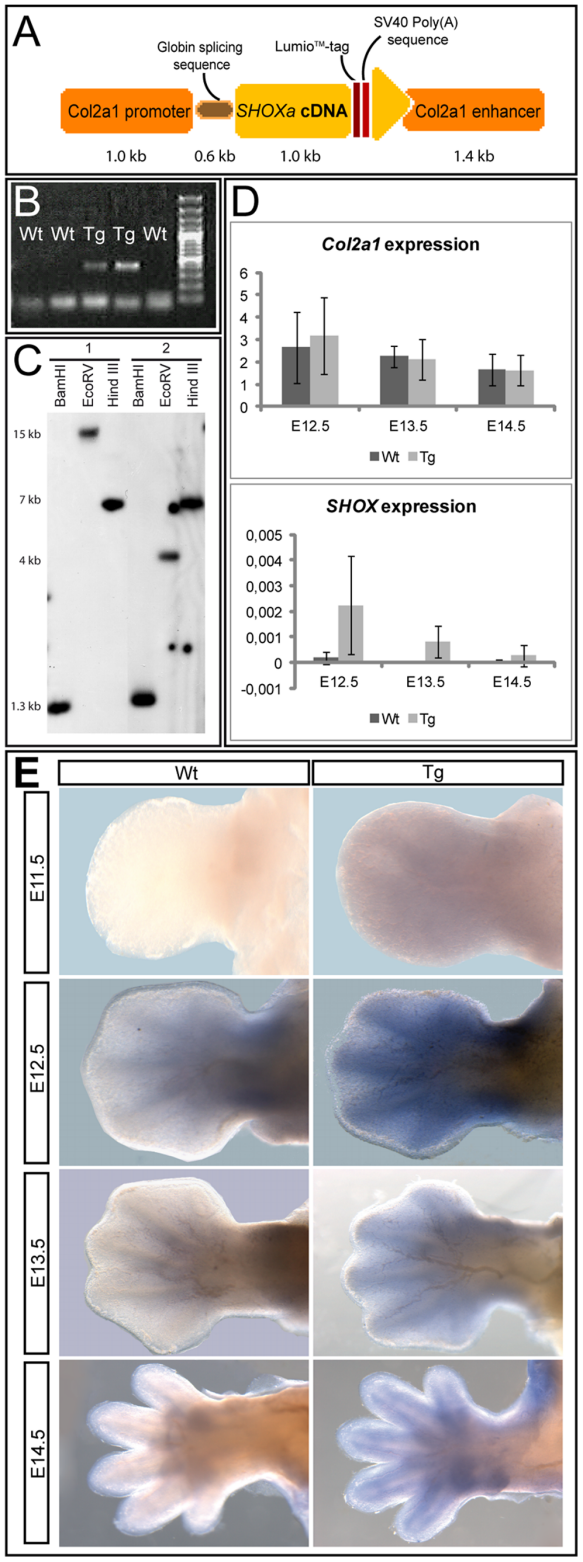
Generation and expression analysis of *SHOX*-transgenic mice. (A): The *SHOXa* cDNA was tagged with a Lumio and SV40 Poly(A) sequence and cloned under the control of a murine *Col2a1* promotor/enhancer expression cassette. (B): Genotyping was performed using specific primers spanning the first 409 nucleotides of the *SHOXa* cDNA. No PCR product was detected in wildtype animals. (C):-Southern Blot analysis of the two transgenic lines (1 and 2) used for our investigations. Genomic DNA was digested with *BamHI*, *EcoRV* and *Hind III*. *BamHI* digestion results in a 1.3 kb fragment that corresponds to the Lumio/SV40-tagged *SHOX* cDNA, which was flanked by *BamHI* sites. The presence of only one signal per lane indicates a single integration site of the transgene. (D): Relative quantitative expression of *Col2a1* and *SHOXa* transcripts in limbs of wildtype and transgenic littermates (N = 5–8 per litter) at E12.5, E13.5 and E14.5. The expression of the transgene corresponds to the expression dynamics of *Col2a1*. *SHOX* levels are generally low with highest expression at E12.5. Values are variable among individual animals as indicated by the standard deviation (SD). (E): WISH of wildtype (Wt) and transgenic (Tg) embryonic limbs from E11.5-E14.5 (N = 20 for each stage). The transgene is weakly expressed in the developing limb at E11.5 and becomes defined around the cartilaginous anlagen at E12.5. From E13.5 onwards, the expression is mainly seen in the mesenchyme around the developing cartilage and in the perichondrium and decreases during later stages.

To generate transgenic mice expressing the human *SHOX* gene, the *SHOXa* coding sequence was cloned into a murine transgene expression vector harbouring the rat *Collagen type II* (*Col2a1*) promoter and enhancer sequence (Fig. 1A). This system was previously used to drive the expression of transgenic constructs in proliferating chondrocytes [27], [28], [29]. Transgenic founders were identified by the presence of the construct *Tg*(*Col2a1-SHOX*) using PCR and were mated with C57Bl/6 mice (Fig. 1B). Two independent heterozygous transgenic lines were investigated in more detail. Southern blot analysis using genomic DNA from animals of the two transgenic lines showed a single integration locus of the transgenic DNA (Fig. 1C). All transgenic animals were viable and fertile, and the *Tg*(*Col2a1-SHOX*) allele was transmitted according to Mendelian ratios.

Transgenic expression was analyzed by quantitative RT-PCR and whole mount *in situ* hybridization (WISH), demonstrating that *Tg(Col2a1-SHOX)* was expressed in the developing limbs (Fig. 1D–E). The expression started from E11.5 onwards (Fig. 1E) with a variable expression level among different transgenic mice. Following the expression dynamics of the endogenous *Col2a1*, *Tg*(*Col2a1-SHOX*) quantities were highest at around E12.5 and gradually decreased during later stages of embryonic development (Fig. 1D). The expression pattern of *Tg*(*Col2a1-SHOX*) at E12.5 resembled *Col2a1* expression which is transcribed at high levels in chondrogenic tissues [32] (Fig. 1E). During later embryonic stages (e.g. E14.5), transgenic expression was confined to the region around the developing cartilage including the perichondrium (Fig. 1E). Thus, the detected expression pattern of the *SHOX*-transgene was comparable to the endogenous *SHOX* expression domains reported in the developing limbs of human and chick embryos [33], [34].

### Analysis of skeletal parameters in *Col2a1-SHOX-*transgenic mice

**Figure 2:**
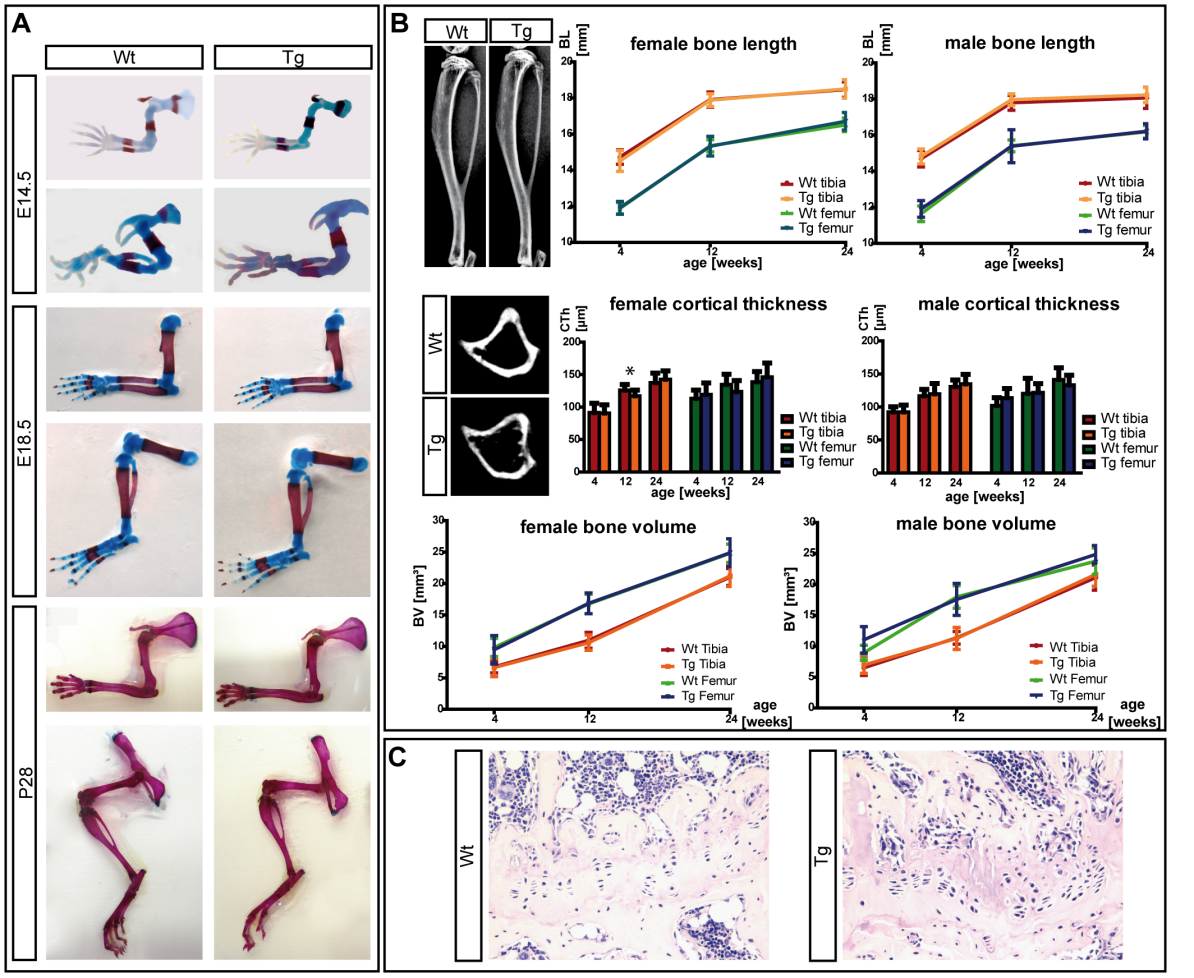
Analysis of postnatal bone parameters of *Col2a1-SHOX*-transgenic mice. (A): Alcian Blue/Alizarin Red S staining at different developmental (E14.5, E18.5) and postnatal (P28) stages does not reveal apparent differences between transgenic and wildtype skeletal elements. (B): Postnatal *in vivo* time-course analysis of bone growth in 65 animals of two transgenic lines by μ-CT analysis. Tibiae and femora of wildtype and *Tg(Col2a1-SHOX)* littermates at the age of 4, 12 and 24 weeks were scanned, female and male individuals were evaluated separately. Total bone length, cortical bone thickness and bone volume do not show significant differences between wildtype and transgenic females or males. Some transgenic animals presented longer bones and weaker structures of the cortical bone in the subcartilaginous region (indicated in the μ-CT images). Other micromorphological parameters (bone mineral density (BMD), trabecular volume and thickness) showed no significant differences. Statistical analyses were performed using student's t-test. (C): hematoxilin and eosin (H&E) stainings of the growth plate in wildtype and transgenic tibiae. Consistent differences between wildtype and *Tg(Col2a1-SHOX)* adult growth plates (24 weeks of age) did not exist (N = 8), but some transgenic tibiae showed a buckling, and the columns of chondrocytes became shorter and were not strictly oriented in a parallel assembly compared to the wildtype (right image).

Transgenic animals showed no obvious difference compared to their wildtype littermates. To investigate whether the *Col2a1-SHOX*-transgene has an effect on embryonic cartilage and bone development, E14.5 and E18.5 embryos were stained with Alcian Blue/Alizarin Red S (Fig. 2A). The transgenic embryos were indistinguishable from wildtype littermates at these stages, indicating that bone formation was grossly normal. As some phenotypic features in patients with SHOX deficiency (e.g. Madelung deformity) are sometimes not detectable before the onset of puberty [4], we also investigated the skeletal elements at postnatal stage P28. Again, no striking phenotype was detected in the transgenic animals (Fig. 2A).

To determine if bone length is increased in transgenic animals, we measured the postnatal bone length in 65 animals of two transgenic lines *in vivo* using micro-computed tomography (μ-CT), which enabled the analysis of different bone-specific parameters simultaneously (Fig. 2B). Tibiae and femora of anaesthetized wildtype and *SHOX*-transgenic mice were scanned *in vivo* in a time-course until 24 weeks of age. Data from female and male mice were analyzed separately to eliminate gender-specific effects. Even though we observed increases in bone length in some transgenic animals, these were not significant (Student's t-test). Significant differences in bone volume and bone mineral density were not found either, indicating that long bone development was largely normal upon *Tg*(*Col2a1-SHOX*) expression. A statistically significant decrease of the cortical bone thickness (CTh) was identified in 12 weeks old female transgenic mice, but not in males or at any other time points. Since the assessment of the growth plate in patients with LWD previously demonstrated a normal to disorganized morphology including abnormal chondrocyte stacking [17], we analyzed the femoral and tibial growth plate morphology of transgenic and wildtype mice (24 weeks of age) using hematoxylin and eosin (H&E), periodic acid-Schiff (PAS) and Masson's trichrome (MT) stainings. In some cases, a buckling of the growth plate was observed, and the columns of chondrocytes became shorter and were not strictly oriented in a parallel assembly (Fig 2C). However, these alterations were not consistently found in all transgenic samples.

### Target gene expression and microarray analyses in *Col2a1-SHOX*-transgenic mice

We performed expression analysis of cartilage- and bone-specific markers from E11.5 to E14.5 using whole mount *in situ* hybridization (WISH) to identify whether limb specific markers show aberrant expression in the *Tg(Col2-SHOX)* embryos (Fig. S1A). We found that early genes such as *Shh* were not altered in the transgenic embryos, indicating that limb initiation and limb bud outgrowth were grossly normal. The expression of *Col2a1, Shox2, Runx2, Ihh* as well as *Col10a1* was similar in transgenic and wildtype embryos, suggesting that chondrocyte proliferation and maturation were largely unaffected. The expression levels of these marker genes were also quantified by qRT-PCR, but no significant differences in the amount of the respective transcripts could be detected.

A regulatory effect of *SHOX* on *FGFR3*, *AGC1* and *NPPB* (*BNP*) was recently reported using human cell lines [20], [24], [26]. We therefore analyzed whether the *SHOX*-transgene was able to alter the expression of the mouse *Fgfr3, Agc1* and *Nppb* genes. By using reversely transcribed RNA from E12.5-E14.5 wildtype and transgenic limbs, we detected no effect on *Fgfr3*, but an increasing effect on *Agc1* (in all three tested stages) and *Nppb* (at E13.5) (Fig. S1B). The finding that *Fgfr3* did not respond to *SHOX*-transgenic expression in mouse is consistent with the fact that the relevant SHOX-regulatory elements in the human *FGFR3* promoter do not exist in mouse, while they are present in *Agc1* and *Nppb*.

**Figure 3:**
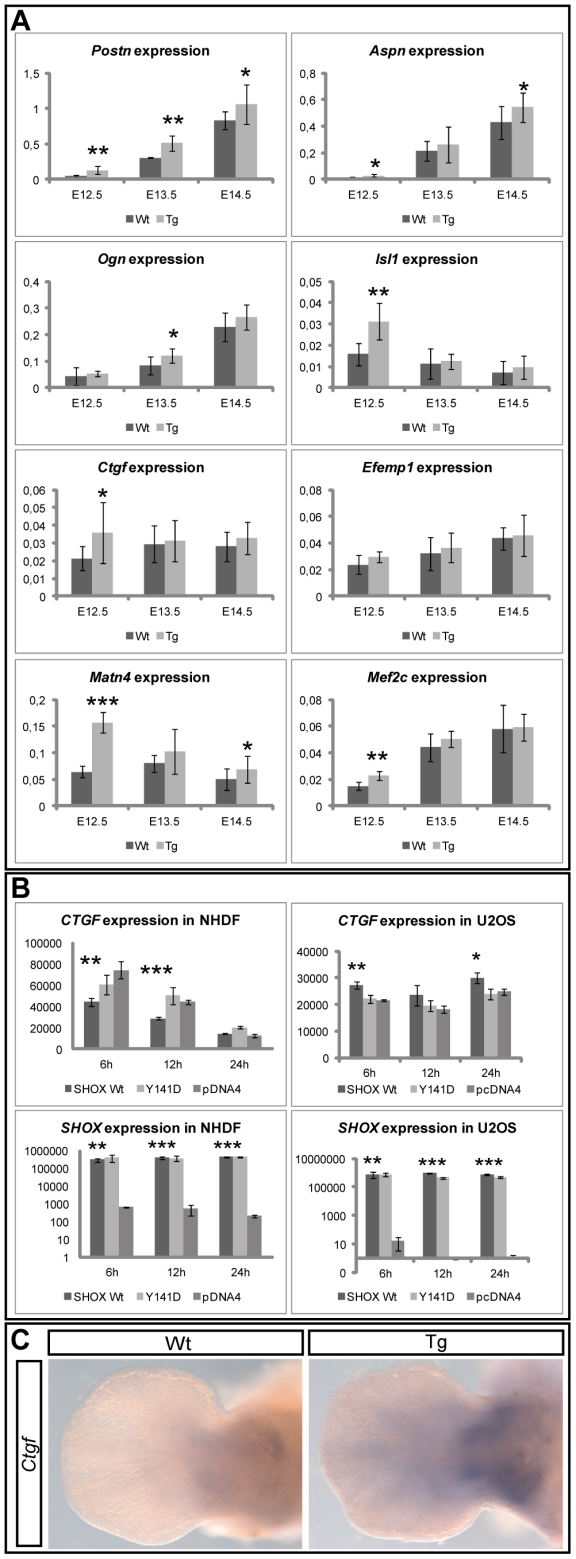
Regulated genes in transgenic mice and validation of *Ctgf* as a target. (A): qRT-PCR using limb RNA (E12.5-E14.5) from wildtype (Wt) and transgenic littermates (Tg) (N = 8–10 for each stage). Measurements were carried out individually, in duplicates, and normalized to *Adam9* and *Sdha*. Relative normalized values are presented on the y-axis. Significances are indicated in each diagram by asterisks (*: *p*≤0.05, **: *p*≤0.01, ***: *p*≤0.001). Variations are indicated by the standard deviation (SD). In 7/8 candidates an upregulation was confirmed as significant in at least one embryonic stage. (B): nCounter analysis of *CTGF* and *SHOX* expression in NHDF and U2OS cells after transient transfections of *SHOX* and *p.Y141D*. *CTGF* is significantly downregulated in NHDF cells, whereas it is significantly upregulated in U2OS cells. Values on y-axis represent absolute counts of mRNA, normalized to *ADAM9*, *HPRT1* and *SDHA*. Significancies are indicated by asterisks. (C): *In situ* hybridization using a *Ctgf* antisense riboprobe on embryonic limbs from wildtype and *SHOX*-transgenic littermates (N = 8) at stage E12.5. In transgenic embryos, enhanced and distalized expression of *Ctgf* was detected in the middle part of the developing limbs.

The altered expression of two known SHOX target genes in transgenic mice prompted us to perform microarray analyses of wildtype and transgenic limb RNA. Prior to hybridization, *Tg*(*Col2a1-SHOX*) expression was confirmed by qRT-PCR and pooled whole limb RNA of either E12.5 wildtype or transgenic littermates were hybridized to microarrays. Selection of differentially regulated genes was carried out using a significant change of expression in both experiments (log2f>0.2 or <-0.2 and *p*<0.05). According to these criteria, 189 genes (83%) were upregulated and 40 genes (17%) were downregulated, suggesting that the *Col2a1*-driven *SHOX*-transgene mainly exerted activating effects. A categorization of differentially expressed genes was performed by gene ontology-based pathway analysis and the most significantly regulated genes were identified in biological pathways associated with either the extracellular matrix or skeletal muscle. The eight most significantly upregulated candidate genes (*Postn*, *Aspn*, *Ogn*, *Isl1*, *Ctgf*, *Efemp1*, *Matn4*, *Mef2c*) that were either known to be involved in limb development, extracellular matrix or skeletal muscle pathways are summarized on Table S1 in File S1. qRT-PCR of the candidate genes was carried out using RNA from wildtype and transgenic limbs of stages E12.5-E14.5. An increase in expression of all candidate target genes including *CTGF* was detected in the transgenic embryos (Fig. 3A).

To further confirm the regulatory effects of SHOX on these genes, we carried out transient transfections of wildtype *SHOX* and a *SHOX* mutant (Y141D) in human U2OS and NHDF cell lines which have been previously used for the characterization of target genes [20], [24], [26]. The p.Y141D variant was identified in two short stature patients and functionally characterized as a defective SHOX protein [10]. For subsequent expression analysis, we applied the nCounter technology that allows direct RNA quantification without reverse transcription into cDNA, resulting in sensitive and reliable detection of mRNA expressed at low abundance. Since the effect of SHOX on validated genes differed between U2OS and NHDF cells, we concluded that the SHOX transcriptional regulation is strongly cell type-dependent (Fig. S2). Most strongly and significantly regulated was the chondrogenic matrix gene *CTGF*, which showed a reduced expression upon *SHOX*-transfections in NHDF and an increased expression in U2OS cells (Fig. 3B). *In situ* hybridization of *Ctgf* on wildtype and *Tg(Col2a1-SHOX)* embryonic limbs showed an increased and a more distal expression in the transgenic limbs (Fig. 3C).

### The connective tissue growth factor gene *CTGF* represents a target of SHOX transactivating functions

**Figure 4:**
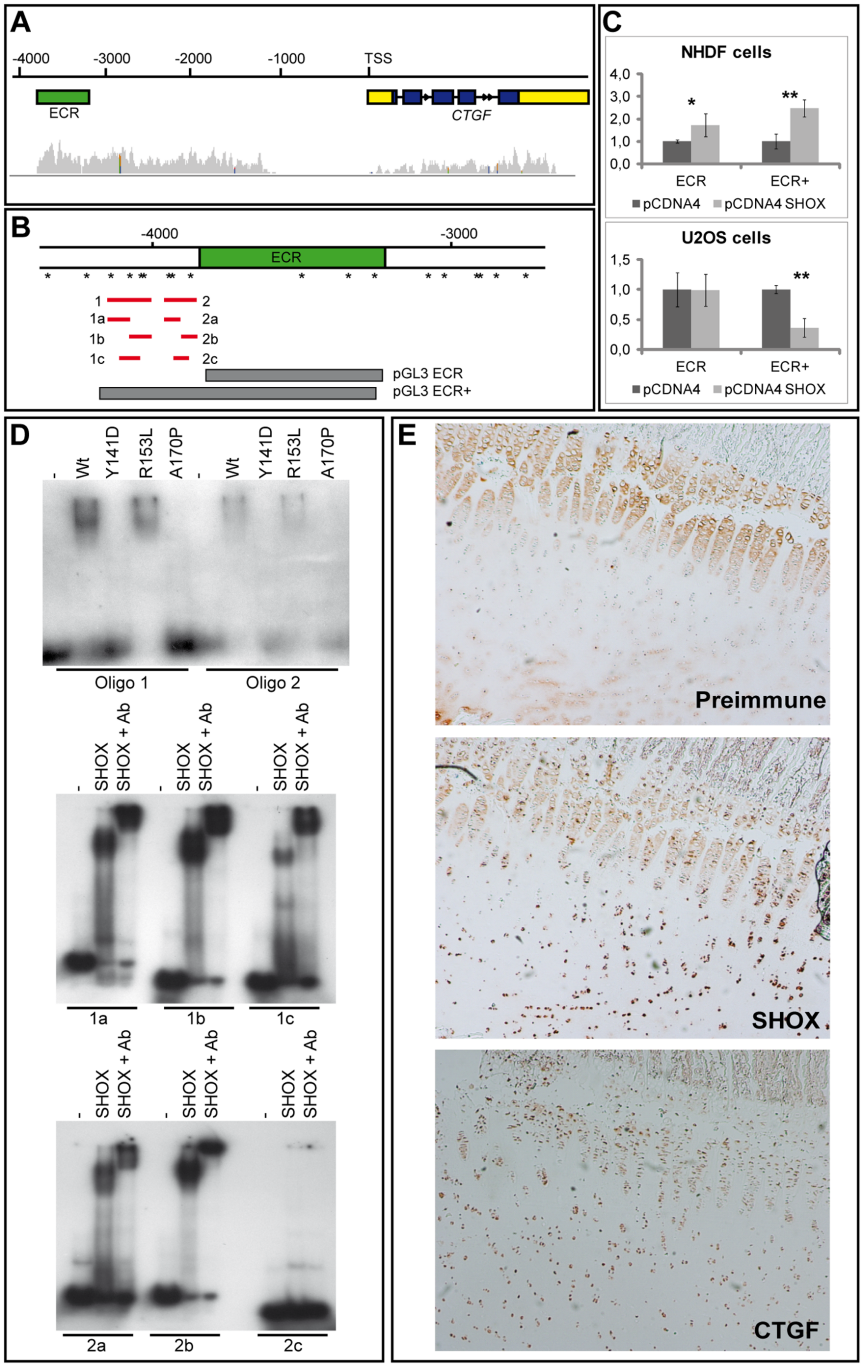
Analysis of *CTGF* as a direct transcriptional target of SHOX. (A): Genomic structure of the human *CTGF* region. ChIP-Seq analysis in ChMM cultures revealed an accumulation of Shox binding in the *Ctgf* promoter region (grey peaks), especially in a region 3–4 kb from the transcriptional start site (TSS) where an evolutionary conserved sequence (ECR) of 597 bp (human chr6:132317086-132318077) was identified (green bar). (B): Location of the pGL3 ECR and pGL3 ECR+ reporter constructs (grey bars) within the *CTGF* upstream region. The ECR+ construct encompasses the ECR and an upstream region including ATTA/TAAT motifs and palindromes. SHOX binding motifs (ATTA/TAAT sites and palindromes) in the *CTGF* 5′ region around the ECR are indicated by asterisks. Red bars represent the location of the generated oligonucleotides for EMSA. (C): Luciferase reporter gene assays in NHDF and U2OS cells. pcDNA4/TO *SHOX* was cotransfected with a luciferase reporter vector harbouring either the ECR or the ECR+ sequence. Transfections and measurements were carried out in triplicates. A significant activation in the luciferase activity was observed 24 h after *SHOX* transfection in NHDF cells using both reporter constructs (1.7-fold/2.5-fold with *p* = 0.02/0.007 for ECR/ECR+). In U2OS cells, an alteration was not observed for the ECR reporter, but a significant reduction was demonstrated for the ECR+ reporter construct (1.0-fold/2.8-fold with *p* = 0.1/0.003 for ECR/ECR+). (D): EMSA. The SHOX wildtype (Wt) and the mutant p.R153L proteins bind to oligonucleotides 1 and 2, whereas the defective proteins p.Y141D and p.A170P cannot. All fragments of oligonucleotides 1 and 2 containing an ATTA/TAAT site are sensitive to SHOX binding (1a–c, 2a–b). The fragment lacking this motif does not bind (oligonucleotide 2c). Using the SHOX-3 antibody (Ab), we demonstrate that the binding is SHOX-specific. (E): Immunohistochemistry performed on pubertal tibial growth plates. Staining was performed using preimmune serum as a negative control, SHOX antibody [19] and a CTGF-specific antibody. Both the SHOX and CTGF proteins were detected in growth plate chondrocytes.

Analyses of the *SHOX*-transgenic mouse and human cell lines overexpressing *SHOX* have demonstrated a regulatory effect of SHOX on *Ctgf/CTGF* expression. In addition, previous ChIP-Seq data on chicken micromass cultures transduced with RCAS-Shox [26] suggest *Ctgf* as a putative cell target of SHOX with several binding sites identified within the 5′ region of the gene. Computational analyses of the human *CTGF* upstream region (5 kb) identified more than 40 binding motifs of the ATTA/TAAT type which have been reported to be the target sites of SHOX [8], [26]. Of these, eight motifs were arranged as palindromes. Furthermore, the region with the highest ChIP-Seq reads in the chicken *Ctgf* locus includes an ECR (evolutionary conserved region) that is also present in the human *CTGF* upstream sequence (Fig. 4A). To demonstrate that *CTGF* could be directly targeted by SHOX, we performed luciferase reporter gene assays in NHDF and U2OS cells. We used two constructs: the smaller one included the human ECR sequence (ECR) and the larger construct included the ECR as well as putative SHOX binding sites (ECR+) (Fig. 4B). As shown in Fig. 4C, significant regulatory effects of SHOX on the ECR+ reporter constructs were observed in both NHDF and U2OS cell lines, whereas for the ECR reporter construct a significant regulation could only be demonstrated in NHDF cells. To confirm a direct binding of SHOX to the *CTGF* upstream region, electrophoretic mobility shift assays (EMSA) were carried out using two oligonucleotide sequences of the ECR+ construct (Oligo 1 and Oligo 2) encompassing the ATTA/TAAT motifs (Fig. 4B). As controls, mutant SHOX proteins (p.Y141D, p.R153L and p.A170P; previously detected in patients with short stature) were used [10]. While the wildtype SHOX and p.R153L proteins bound to the tested sequences, p.Y141D and p.A170P did not (Fig. 4D). Further subdivision of oligonucleotides 1 and 2 narrowed down SHOX binding to all fragments where ATTA/TAAT sites were present (Fig. 4B and 4D). To demonstrate physiological relevance of these data, immunohistological staining on sections from human pubertal growth plate specimen were carried out. Using CTGF and SHOX specific antibodies, coexpression was detected in hypertrophic chondrocytes (Fig. 4E).

## Discussion

### Generation and expression studies of *Col2a1-SHOX*-transgenic mice

For a small number of human protein-coding genes, a mouse ortholog does not exist [35]. One approach to learn more about the biology of these human genes is to introduce them into mice. We have generated transgenic mice that express the human *SHOX* cDNA in embryonic limbs under the control of the murine *Col2a1* promoter/enhancer. Expression of the *SHOX*-transgene was detected between E12.5 and E14.5. Compared to *Col2a1*, a highly abundant major structural component of the extracellular matrix, the expression of the transcription factor *SHOX* was very weak and differed between animals. The generation of a transgenic mouse using a different promoter and/or enhancer may eventually yield in higher *SHOX* expression levels. However, low expression levels are characteristic for *SHOX* and have been found in all tissues and cell lines tested [22], suggesting that SHOX functions do not rely on high mRNA or protein abundance in the cell.

### Analysis of skeletal parameters

Phenotypic analyses of the developing limbs in transgenic mice did not reveal significant differences compared to wildtype (with the exception of cortical thickness in female tibiae at 12 weeks and almost significant differences in female femora). Thus, there may be gender-specific effects in the transgenic mice during postnatal growth, however, to address this question, more detailed experiments would be necessary. Phenotypic clinical features have been previously assessed in patients with isolated SHOX deficiency and LWD [6], but not much data on cortical bone structures, bone volume or mineral density is available. Patients with Turner syndrome (45,X) suffer from a high fracture risk and have reduced cortical bone structures and bone mineral density [36], but whether this is due to reduced *SHOX* expression is not known. Disorganization of the growth plate has been noted in some of our *SHOX*-transgenic mice, but is not a consistent feature. Disturbed growth plate morphology has been described in patients with LWD [17], but no data is available on patients with additional *SHOX* copies.

### Gene expression and microarray analyses

To determine if the critical stages in endochondral ossification were altered in the transgenic mice, we carried out expression analysis of embryonic limb marker genes and could demonstrate that expression of these genes remained intact. A key question also concerned the extent to which the human gene is correctly read by the mouse transcriptional machinery. We therefore tested expression of all three known SHOX target genes [20], [24], [26] and obtained elevated mean expression levels for *Agc1* and *Nbbp* as expected, probably due to the conserved SHOX-sensitive binding sites in the *Agc1 and Nppb* enhancer and promoter regions, while the human SHOX-sensitive binding sites in the *Fgfr3* promoter do not exist in mouse.

To further search for effects of the *SHOX*-transgene, we carried out microarray analysis and identified many regulated genes belonging to the extracellular matrix and skeletal muscle pathways. It is interesting that several of these genes, including *Postn* and *Matn4*, have been previously also identified as targets in *Shox2*-deficient mice and thus may represent targets for both SHOX and Shox2 [37]. The mouse Shox2 protein is 79% identical to human SHOX and their 60 amino acid binding domains (the homeodomain) are identical [33]. *In situ* analysis have demonstrated a more proximal expression domain of the *SHOX* paralog *SHOX2* in human and also in chick embryonic limbs [33], [34], and conditional deletion of *Shox2* in the developing mouse limbs dramatically impairs the formation of the proximal limb elements [38], [39]. A substitution of the *Shox2* locus by human *SHOX* in mouse has demonstrated that *SHOX* is able to ameliorate but not to fully rescue *Shox2*-deficient limb anomalies, suggesting only partial functional redundancy [25].

We have selected eight putatively regulated genes (*Postn*, *Aspn*, *Ogn*, *Isl1*, *Ctgf*, *Efemp1*, *Matn4*, *Mef2c*) for further analysis and could demonstrate a significant deregulation in E12.5-E14.5 *SHOX*-transgenic limbs compared to wildtype in seven of the eight genes. To further validate these candidates, we also tested them in NHDF and U2OS cells and 5/8 (NHDF) and 4/8 (U2OS) were shown to be significantly regulated in these human cells. Taken together, our data demonstrate that the identified target genes of *Tg(Col2a1-SHOX)* are SHOX-specific and do not represent transgenic artifacts. It is also reassuring that the human *SHOX* is expressed in the appropriate stage- and cell-type specific manner in mouse and we confirm previous data that SHOX can act both as an activator and repressor of target genes in a cell-type specific fashion [25], [26].

### *CTGF* represents a direct SHOX target gene

Quantitative analyses in mouse and human cells identified *CTGF*/*Ctgf* as the most consistently regulated candidate target gene. Enhanced and slightly distalized expression of *Ctgf* was also seen at E12.5 (the stage of highest *SHOX* expression) in transgenic mouse limbs using WISH. Further evidence for *Ctgf* as a target of Shox was derived from ChIP-Seq data in chicken which identified several Shox binding sites in the *Ctgf* upstream region. Multiple SHOX binding motifs and an ECR were identified, and by luciferase and EMSA experiments, we could show that the extended ECR region (ECR+) is responsive to SHOX in human cells. The finding that the *CTGF* mRNA was either down- (NHDF) or upregulated (U2OS) indicates a complex transcriptional regulation. The remarkable accumulation of Shox-mediated reads (respective binding sites) identified in the chicken *Ctgf* upstream region using ChIP-Seq (Fig. 4A) suggests that additional response elements outside the ECR may also be sensitive to SHOX, and these, together with a spatio-temporal composition of cofactors, may contribute to the fine regulation of *CTGF* expression in a given cellular environment. Physiological relevance of the SHOX-*Ctgf/CTGF* relationship is suggested by the coexpression of both, SHOX and CTGF proteins in hypertrophic chondrocytes of the human growth plates.

According to its expression pattern, *SHOX* deficiency results in shortening and deformation of radii/ulnae and tibiae/fibulae. Comparable to *SHOX* deficiency, the skeletal defects in *Ctgf* null mice are also specific for radii/ulnae and tibiae/fibulae and not for the proximal elements of the limbs [40]. Interestingly, the phenotypes of *SHOX*- as well as *Ctgf*-*transgenic* mice [41] are less severe than the loss-of-function phenotypes and strongly dependent on the expression level of the transgenes. Even though *Ctgf*-transgenic mice show more stigmata than *SHOX*-transgenic individuals, phenotypic differences were reported only at postnatal stages and also include cortical thickness and *Agc1* expression [41]. Since *Agc1* has been found to be reduced in *Ctgf* mutant mice [40] and to be regulated by SHOX in human cells [20], the demonstrated regulation may be indirect and mediated through *Ctgf*. This is also supported by our finding that the response of *CTGF* is an immediate consequence following *SHOX* overexpression, whereas the regulation of *AGC1* occurs at a later time point (Fig. S2). *Ctgf* null mice suffer from multiple defects, such as failure in growth plate chondrogenesis, angiogenesis, extracellular matrix production and bone formation/mineralization [40]. A role of SHOX during angiogenesis has been speculated, since *Shox* expression was detected in the vasculature of the developing chicken limbs [34]. However, a contribution of SHOX in other *CTGF*-associated conditions such as fibrotic disease, inflammation and cancer [42], [43], [44] is not known.

In summary, we have established a transgenic mouse model expressing *SHOX* under the control of the *Col2a1* promoter and enhancer. By combining data from mouse and chicken micromass cultures and human cell culture experiments, we could identify activating or repressing effects of SHOX on target genes, depending on spatio-temporal conditions and cell types. We have also demonstrated a direct regulatory effect on *CTGF* which may take place in the hypertrophic zone of the human growth plate. We have shown a direct binding of the SHOX protein to a highly conserved upstream region of the *CTGF* gene, identified by ChIP-Seq, resulting in regulatory effects in reporter gene assays in human cell lines. Since CTGF is involved in various biological processes, the effect of SHOX on *CTGF* expression in these different processes can now be investigated.

## Supporting Information

Figure S1 Marker and target gene analysis during embryonic development. (A): WISH of limb marker genes from E11.5 to E14.5. At E11.5, when *Tg(Col2a1-SHOX)* expression was first detected in the developing limb, limb buds in transgenic animals were indistinguishable from the wildtype. Expression of the *Shh* morphogen as a marker gene during limb initiation and outgrowth was normal. Also at E12.5 when *Tg(Col2a1-SHOX)* is most prominently expressed, chondrocyte proliferation in the transgenic animals appeared normal, as represented by *Col2a1* expression comparable to the wildtype. Also, the *SHOX*-homologue *Shox2* and its downstream gene *Runx2* were normally expressed in *SHOX*-transgenic animals at E12.5. *Runx2* is known to regulate chondrocyte maturation and *Ihh* expression, which was also unaffected in *Tg(Col2a1-SHOX)* limbs at E13.5. Following chondrocyte proliferation at E14.5 in both wildtype and transgenic embryos, a specific *Col10a1* pattern is detected which defines chondrocyte hypertrophy. (B): Quantitative RT-PCR on embryonic limb RNA of stages E12.5–E14.5 using primers for the SHOX target genes *Fgfr3*, *Agc1* and *Nppb*. cDNA of wildtype and transgenic littermates of each stage (N = 8–12) were measured individually and in duplicates. Measurements were normalized to *Adam9* and *Sdha*; values on y-axis represent relative normalized expression. The expression of *Fgfr3* was unaltered in transgenic limbs. Mean *Agc1* expression was increased during E12.5 and E13.5, a trend which did, however not reach significance (E12.5: 2.0-fold, *p* = 0.068; E13.5: 2.6-fold, *p* = 0.092; E14.5: 1.3-fold, *p* = 0.377). *Nppb* expression levels were weakly increased at E13.5 (1.7-fold, *p* = 0.104).
(TIF)

Figure S2 nCounter analysis of eight selected candidate genes in NHDF and U2OS cells. RNA was isolated 6 h, 12 h and 24 h after transfection of expression constructs for SHOX, SHOX Y141D (a defective SHOX variant (1)) and a control (pCDNA4). Measurements were carried out in triplicates and normalized to *ADAM9, HPRT1* and *SDHA*. As a control, *SHOX* expression upon its target gene *AGC1* was analyzed. Upon strong increase of *SHOX*, *AGC1* was significantly activated 12 hours after *SHOX*-tranfection. Values on y-axis represent absolute counts of mRNA. Significancies of the *SHOX*-transfected samples are indicated in each diagram by asterisks. *: *p*≤0.05, **: *p*≤0.01, ***: *p*≤0.001.
(TIF)

File S1 Contains Table S1, Genes, gene characterization, fold regulation and p-values of eight selected upregulated genes in the microarray. Table S2, Primers, Probes and Oligonucleotides.
(DOC)
