# Supplementary figures and images for: Identification of Novel SHOX Target Genes in the Developing Limb Using a Transgenic Mouse Model
Source: PLoS One. 2014 Jun 2;9(6):e98543. doi: 10.1371/journal.pone.0098543 (PMC4041798; doi:10.1371/journal.pone.0098543)

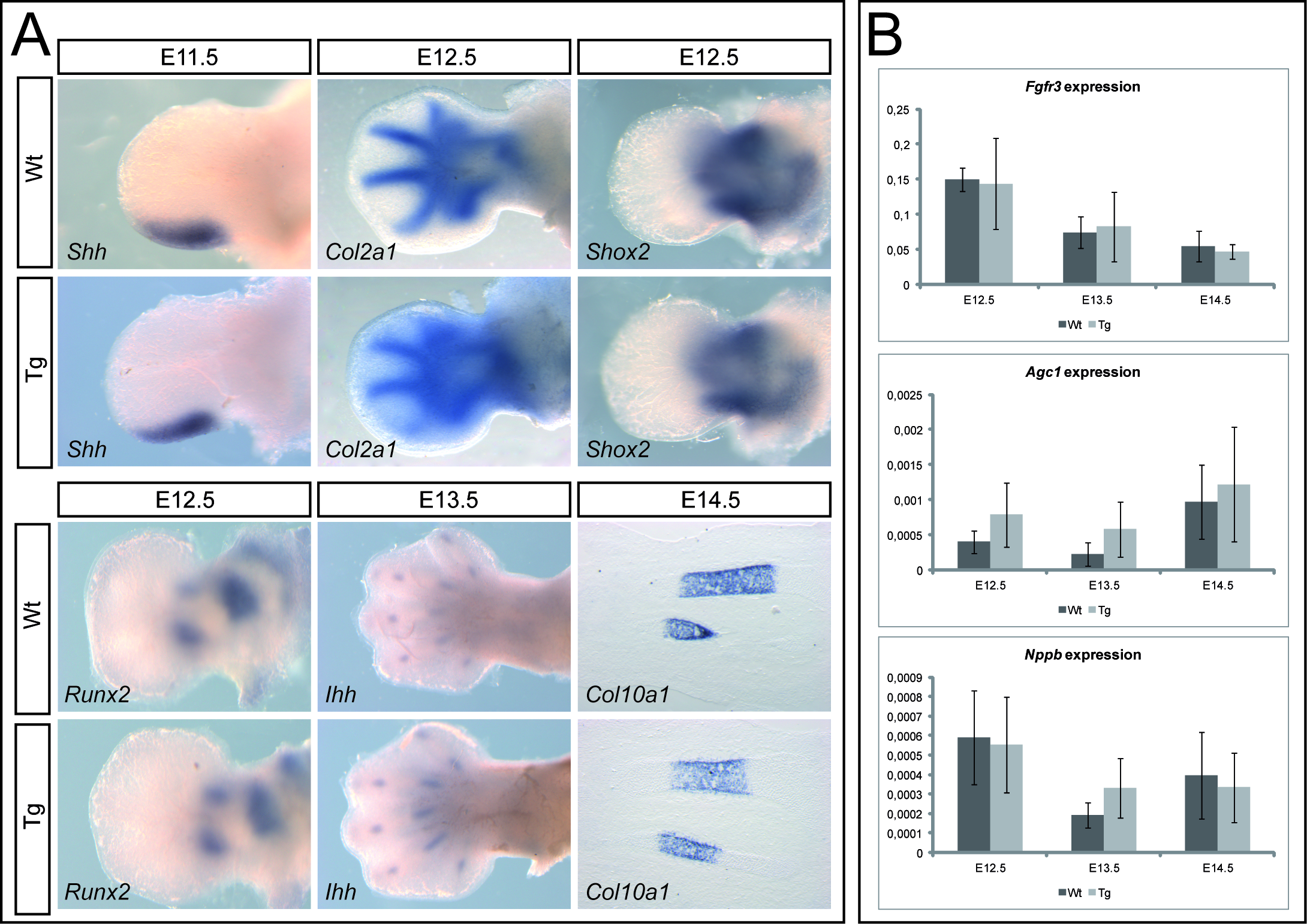

Supplement: Figure S1 — Marker and target gene analysis during embryonic development. (A): WISH of limb marker genes from E11.5 to E14.5. At E11.5, when Tg(Col2a1-SHOX) expression was first detected in the developing limb, limb buds in transgenic animals were indistinguishable from the wildtype. Expression of the Shh morphogen as a marker gene during limb initiation and outgrowth was normal. Also at E12.5 when Tg(Col2a1-SHOX) is most prominently expressed, chondrocyte proliferation in the transgenic animals appeared normal, as represented by Col2a1 expression comparable to the wildtype. Also, the SHOX-homologue Shox2 and its downstream gene Runx2 were normally expressed in SHOX-transgenic animals at E12.5. Runx2 is known to regulate chondrocyte maturation and Ihh expression, which was also unaffected in Tg(Col2a1-SHOX) limbs at E13.5. Following chondrocyte proliferation at E14.5 in both wildtype and transgenic embryos, a specific Col10a1 pattern is detected which defines chondrocyte hypertrophy. (B): Quantitative RT-PCR on embryonic limb RNA of stages E12.5–E14.5 using primers for the SHOX target genes Fgfr3, Agc1 and Nppb. cDNA of wildtype and transgenic littermates of each stage (N = 8–12) were measured individually and in duplicates. Measurements were normalized to Adam9 and Sdha; values on y-axis represent relative normalized expression. The expression of Fgfr3 was unaltered in transgenic limbs. Mean Agc1 expression was increased during E12.5 and E13.5, a trend which did, however not reach significance (E12.5: 2.0-fold, p = 0.068; E13.5: 2.6-fold, p = 0.092; E14.5: 1.3-fold, p = 0.377). Nppb expression levels were weakly increased at E13.5 (1.7-fold, p = 0.104). (TIF) [file pone.0098543.s001.tif]

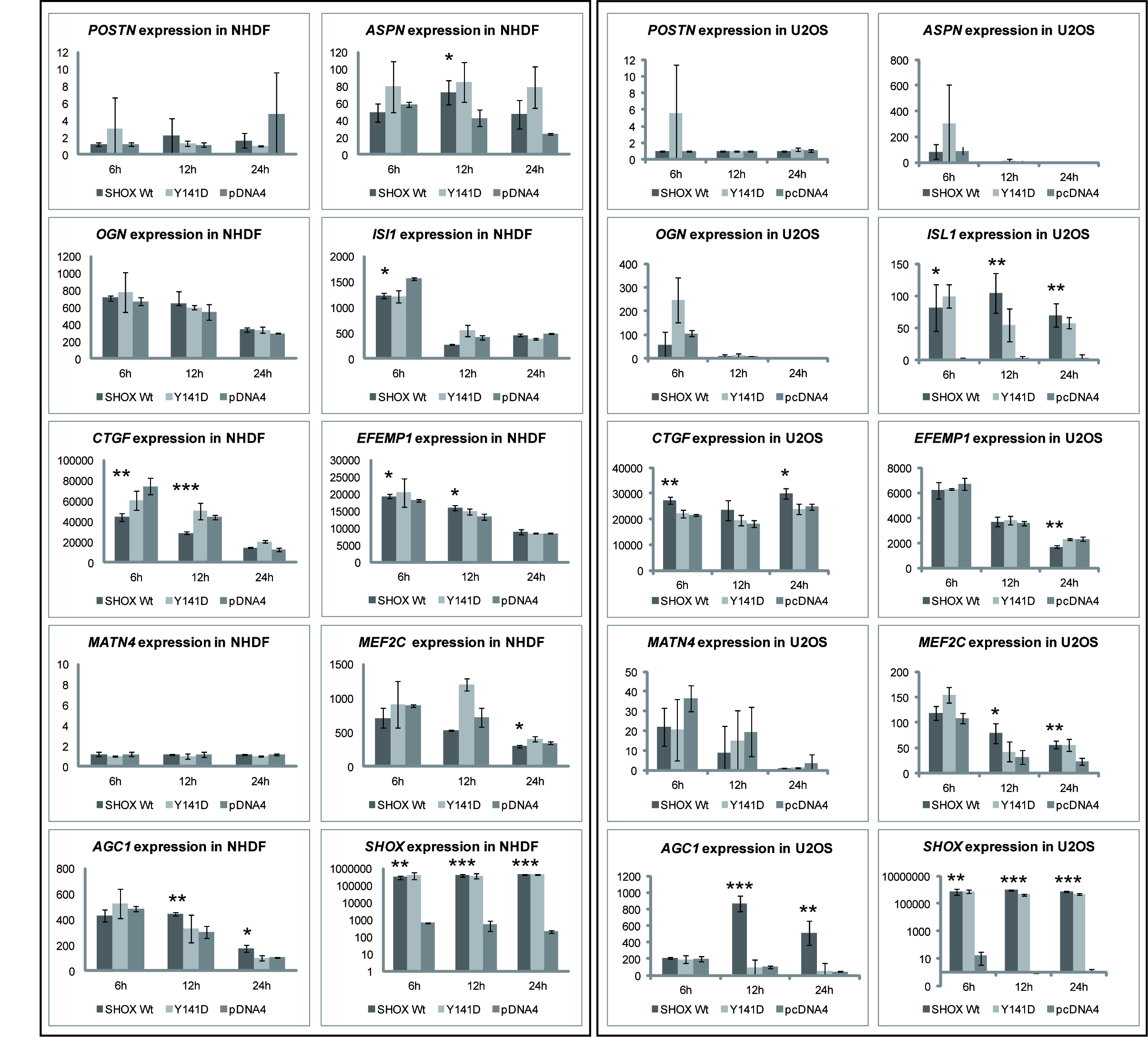

Supplement: Figure S2 — nCounter analysis of eight selected candidate genes in NHDF and U2OS cells. RNA was isolated 6 h, 12 h and 24 h after transfection of expression constructs for SHOX, SHOX Y141D (a defective SHOX variant (1)) and a control (pCDNA4). Measurements were carried out in triplicates and normalized to ADAM9, HPRT1 and SDHA. As a control, SHOX expression upon its target gene AGC1 was analyzed. Upon strong increase of SHOX, AGC1 was significantly activated 12 hours after SHOX-tranfection. Values on y-axis represent absolute counts of mRNA. Significancies of the SHOX-transfected samples are indicated in each diagram by asterisks. *: p≤0.05, **: p≤0.01, ***: p≤0.001. (TIF) [file pone.0098543.s002.tif]
