# Supplementary material for: Identification of Novel SHOX Target Genes in the Developing Limb Using a Transgenic Mouse Model
Source: PLoS One. 2014 Jun 2;9(6):e98543. doi: 10.1371/journal.pone.0098543 (PMC4041798; doi:10.1371/journal.pone.0098543)
Supplement: File S1 — Contains Table S1, Genes, gene characterization, fold regulation and p-values of eight selected upregulated genes in the microarray. Table S2, Primers, Probes and Oligonucleotides. (DOC) [file pone.0098543.s003.doc]

**SUPPORTING INFORMATION**

**SI Figure Legends**

**Fig. S1:** Marker and target gene analysis during embryonic development. (A): WISH of limb marker genes from E11.5 to E14.5. At E11.5, when *Tg(Col2a1-SHOX*) expression was first detected in the developing limb, limb buds in transgenic animals were indistinguishable from the wildtype. Expression of the *Shh* morphogen as a marker gene during limb initiation and outgrowth was normal. Also at E12.5 when *Tg(Col2a1-SHOX)* is most prominently expressed, chondrocyte proliferation in the transgenic animals appeared normal, as represented by *Col2a1* expression comparable to the wildtype. Also, the *SHOX*-homologue *Shox2* and its downstream gene *Runx2* were normally expressed in *SHOX*-transgenic animals at E12.5. *Runx2* is known to regulate chondrocyte maturation and *Ihh* expression, which was also unaffected in *Tg(Col2a1-SHOX)* limbs at E13.5. Following chondrocyte proliferation at E14.5 in both wildtype and transgenic embryos, a specific *Col10a1* pattern is detected which defines chondrocyte hypertrophy. (B): Quantitative RT-PCR on embryonic limb RNA of stages E12.5–E14.5 using primers for the SHOX target genes *Fgfr3*, *Agc1* and *Nppb*. cDNA of wildtype and transgenic littermates of each stage (N=8-12) were measured individually and in duplicates. Measurements were normalized to *Adam9* and *Sdha;* values on y-axis represent relative normalized expression. The expression of *Fgfr3* was unaltered in transgenic limbs. Mean *Agc1* expression was increased during E12.5 and E13.5, a trend which did, however not reach significance (E12.5: 2.0-fold, *p*= 0.068; E13.5: 2.6-fold, *p*= 0.092; E14.5: 1.3-fold, *p*= 0.377). *Nppb* expression levels were weakly increased at E13.5 (1.7-fold, *p*= 0.104).

**Fig. S2**: nCounter analysis of eight selected candidate genes in NHDF and U2OS cells. RNA was isolated 6h, 12h and 24h after transfection of expression constructs for SHOX, SHOX Y141D (a defective SHOX variant (1)) and a control (pCDNA4). Measurements were carried out in triplicates and normalized to *ADAM9, HPRT1* and *SDHA*. As a control, *SHOX* expression upon its target gene *AGC1* was analyzed. Upon strong increase of *SHOX*, *AGC1* was significantly activated 12 hours after *SHOX*-tranfection. Values on y-axis represent absolute counts of mRNA. Significancies of the *SHOX*-transfected samples are indicated in each diagram by asterisks. *: *p*< 0.05, **: *p*< 0.01, ***: *p*< 0.001.

**Table S1:** Genes, Gene characterization, fold regulation and p-values of eight selected upregulated genes in the microarray.

| **Gene name** | **Gene symbol** | **gene characterization** | **fold** | **p-value** | **References** |
| --- | --- | --- | --- | --- | --- |
| *Periostin* | *Postn* | proteinaceous extracellular matrix, cell adhesion, skeletal system development, originally identified in osteblasts and named after its expression in the periosteum, heart development, role in tumorigenesis | 1.48 | 2.90E-07 | 1, 2 |
| *Asporin* | *Aspn* | cartilage small leucine-rich proteoglycan, proteinaceous extracellular matrix, bone mineralization | 1.31 | 3.65E-05 | 3, 4 |
| *Osteoglycin* | *Ogn* | small leucine-rich proteoglycan, proteinaceous extracellular matrix | 1.30 | 2.13E-13 | 4, 5 |
| *Islet 1* | *Isl1* | LIM/homeodomain transcription factor, multicellular organismal development, key regulator for hindlimb initiation | 1.29 | 1.14E-07 | 6 |
| *Connective tissue growth factor* | *Ctgf* | cartilage condensation, angiogenesis, cell-matrix adhesion, coordinator of chondrogenesis and angiogenesis in hypertrophic chondrocytes | 1.26 | 1.11E-06 | 7, 8 |
| *EGF-containing fibulin-like extracellular matrix protein 1* | *Efemp1* | negative regulation of chondrocyte differentiation, epidermal growth factor receptor signaling pathway, GWAS-associated gene for height | 1.26 | 3.55E-05 | 9, 10 |
| *Matrilin 4* | *Matn4* | extracellular region, protein binding | 1.16 | 6.25E-05 | 11 |
| *Myocyte enhancer factor 2* | *Mef2c* | chondrocyte/osteblast/muscle cell differentiation, apoptosis, associated with cardiac and skeletal muscle development, matrix mineralization | 1.13 | 1.00E-04 | 12 |
| Characterization is based on ENTREZ gene summary, AMIGO and references given. Most genes represent extracellular matrix genes. | | | | | |

**Table S2:** Primers, Probes and Oligonucleotides

**SI References**

1. Horiuchi K*, et al.* (1999) Identification and characterization of a novel protein, periostin, with restricted expression to periosteum and periodontal ligament and increased expression by transforming growth factor beta. *J Bone Miner Res* 14(7):1239-1249.

2. Ruan K, Bao S, & Ouyang G (2009) The multifaceted role of periostin in tumorigenesis. *Cell Mol Life Sci* 66(14):2219-2230.

3. Henry SP*, et al.* (2001) Expression pattern and gene characterization of asporin. a newly discovered member of the leucine-rich repeat protein family. *J Biol Chem* 276(15):12212-12221.

4. Tasheva ES, Klocke B, & Conrad GW (2004) Analysis of transcriptional regulation of the small leucine rich proteoglycans. *Mol Vis* 10:758-772.

5. Tasheva ES*, et al.* (2002) Mimecan/osteoglycin-deficient mice have collagen fibril abnormalities. *Mol Vis* 8:407-415.

6. Kawakami Y*, et al.* (2011) Islet1-mediated activation of the beta-catenin pathway is necessary for hindlimb initiation in mice. *Development* 138(20):4465-4473.

7 Dhar A & Ray A (2010) The CCN family proteins in carcinogenesis. *Exp Oncol* 32(1):2-9.

8. Ivkovic S*, et al.* (2003) Connective tissue growth factor coordinates chondrogenesis and angiogenesis during skeletal development. *Development* 130(12):2779-2791.

9. Wakabayashi T*, et al.* (2010) Fibulin-3 negatively regulates chondrocyte differentiation. *Biochem Biophys Res Commun* 391(1):1116-1121.

10. Weedon MN*, et al.* (2008) Genome-wide association analysis identifies 20 loci that influence adult height. *Nat Genet* 40(5):575-583.

11. Klatt AR, Paulsson M, & Wagener R (2002) Expression of matrilins during maturation of mouse skeletal tissues. *Matrix Biol* 21(3):289-296.

12. Stephens AS*, et al.* (2011) Myocyte enhancer factor 2c, an osteoblast transcription factor identified by dimethyl sulfoxide (DMSO)-enhanced mineralization. *J Biol Chem* 286(34):30071-30086.
